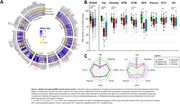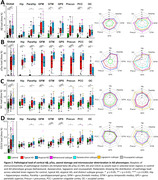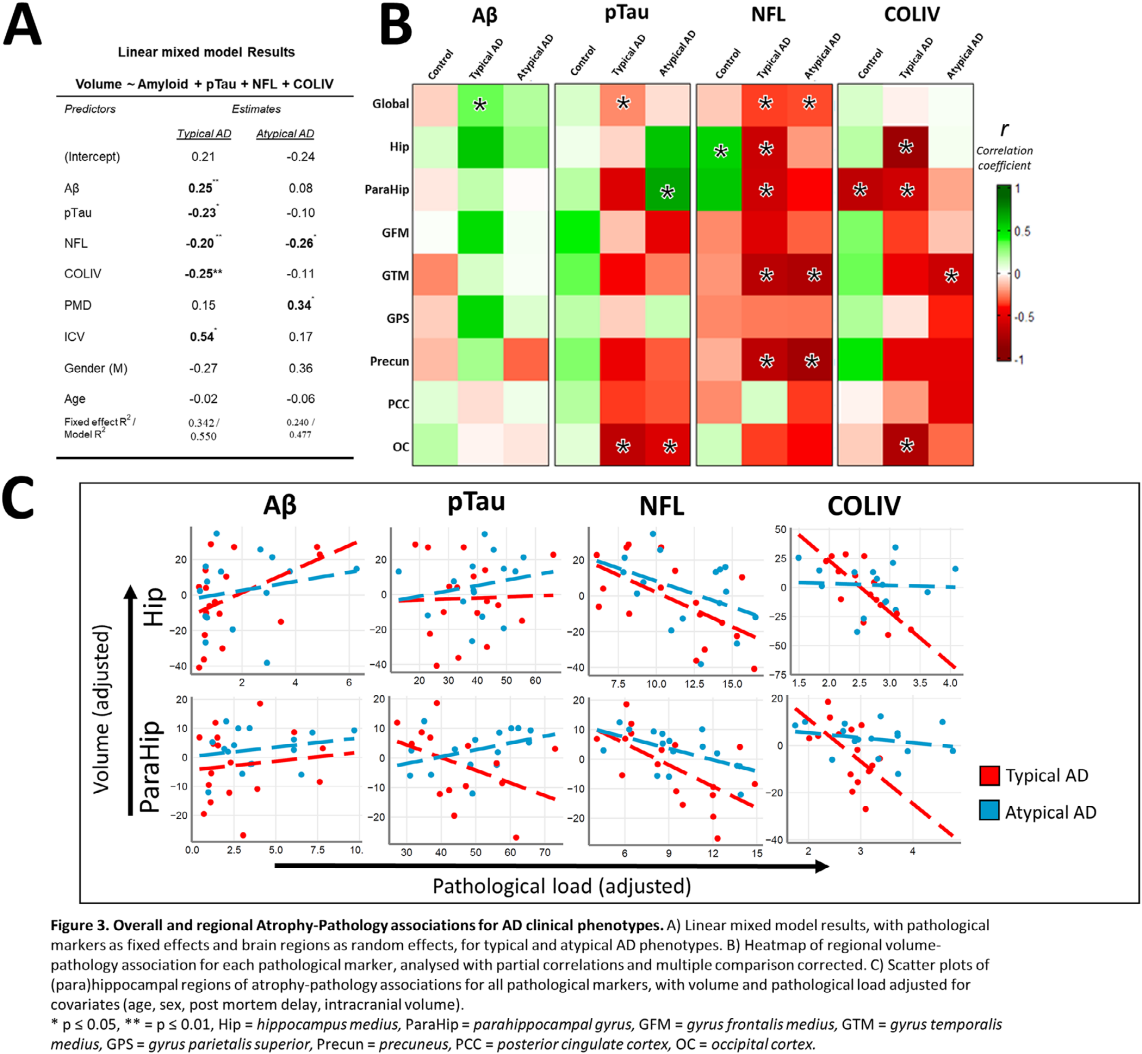# Clinical Phenotypes of Alzheimer’s Disease: Atrophy Patterns and their Pathological Correlates

**DOI:** 10.1002/alz.087358

**Published:** 2025-01-03

**Authors:** Niels Reijner, Irene Frigerio, Maud M.A. Bouwman, Baayla D.C. Boon, Nicolas Guizard, Thomas Jubault, Jeroen J.M. Hoozemans, Annemieke J.M. Rozemuller, Femke H. Bouwman, Frederik Barkhof, Elizabeth Gordon, Wilma D.J. van de Berg, Laura E. Jonkman

**Affiliations:** ^1^ Amsterdam UMC, location VUmc, Department of Anatomy and Neurosciences, Section Clinical Neuroanatomy and Biobanking, Amsterdam Netherlands; ^2^ Mayo Clinic, Jacksonville, FL USA; ^3^ Qynapse, Paris France; ^4^ Department of Pathology, Amsterdam Neuroscience, Amsterdam UMC, Amsterdam, Netherlands, Amsterdam, Netherlands, Amsterdam Netherlands; ^5^ Alzheimer Center Amsterdam, Department of Neurology, Amsterdam Neuroscience, Vrije Universiteit Amsterdam, Amsterdam UMC, Amsterdam Netherlands; ^6^ University College London, London United Kingdom; ^7^ Department of Radiology and Nuclear Medicine, Vrije Universiteit Amsterdam, Amsterdam University Medical Center, location VUmc, Amsterdam Netherlands

## Abstract

**Background:**

Recent studies highlight distinct patterns of cortical atrophy between amnestic (typical) and non‐amnestic (atypical, with subtypes: behavioural, dysexecutive, logopenic and visuospatial) clinical phenotypes of Alzheimer’s disease (AD). The current study aimed to assess regional MRI patterns of cortical atrophy across AD phenotypes, and their association with amyloid‐beta (Aβ), phosphorylated tau (pTau), axonal degeneration (NfL) and microvascular deterioration (COLIV).

**Method:**

Postmortem In‐situ 3DT1 3T‐MRI data was collected for 33 AD (17 typical, 16 atypical) and 16 control brain donors. Images were segmented and AAL3 atlas regional volumes were obtained using QyScore®. At subsequent autopsy, eight brain regions were selected, immunostained for Aβ (4G8), pTau (AT8), Neurofilament‐light (NFL), and Collagen‐IV (COLIV), and quantified using qupath. Group comparisons and volume‐pathology associations were analyzed using linear models and partial correlations with covariates age, sex, postmortem delay, and intracranial volume.

**Results:**

Compared to controls, AD phenotype groups showed overall lower cortical volume, while only minor volume differences were observed between AD phenotype groups, observed primarily in limbic regions (Fig. 1). Across pathological markers, AD phenotype groups showed consistently higher immunoreactivity than controls, while atypical AD showed consistently higher immunoreactivity than typical AD (Fig. 2). Moreover, different patterns of pathology could be observed between atypical subtypes (e.g. distinctly higher pTau load in the occipital gyrus of the visuospatial subtype). In typical AD, global volume loss was associated with lower Aβ and higher pTau, NFL and COLIV immunoreactivity, while in atypical AD, global volume loss was primarily associated with higher NFL immunoreactivity (Fig. 3a). Regionally, AD phenotype differences in atrophy‐pathology association were most pronounced in the (para) hippocampal regions. This distinction was mainly characterized by negative associations for NFL and COLIV, which was only observed in typical AD (Fig. 3b‐c).

**Conclusion:**

Atrophy patterns between AD phenotypes showed only minor differences, potentially attributable to the on average later disease stage of the study cohort. The higher immunoreactivity for pathological markers found in atypical AD might suggest a more severe disease burden. (Para) hippocampal volume decline was associated with axonal and microvascular deterioration in typical AD, but not in the higher pathologically burdened atypical AD group, suggesting a differential susceptibility between AD phenotypes.